# Microflow system promotes acetaminophen crystal nucleation

**DOI:** 10.1002/elsc.202000021

**Published:** 2020-07-14

**Authors:** Akari Nishigaki, Mihoko Maruyama, Munenori Numata, Chisako Kanzaki, Shun‐Ichi Tanaka, Hiroshi Y. Yoshikawa, Masayuki Imanishi, Masashi Yoshimura, Yusuke Mori, Kazufumi Takano

**Affiliations:** ^1^ Department of Biomolecular Chemistry Kyoto Prefectural University Kyoto Japan; ^2^ Graduate School of Engineering Osaka University Suita Japan; ^3^ Department of Chemistry Saitama University Saitama Japan; ^4^ Institute of Laser Engineering Osaka University Suita Japan

**Keywords:** anti‐solvent method, laminar flow, liquid–liquid interface, metastable form, polymorph control

## Abstract

It is known that interfaces have various impacts on crystallization from a solution. Here, we describe crystallization of acetaminophen using a microflow channel, in which two liquids meet and form a liquid–liquid interface due to laminar flow, resulting in uniform mixing of solvents on the molecular scale. In the anti‐solvent method, the microflow mixing promoted the crystallization more than bulk mixing. Furthermore, increased flow rate encouraged crystal formation, and a metastable form appeared under a certain flow condition. This means that interface management by the microchannel could be a beneficial tool for crystallization and polymorph control.

**Abbreviation**
PXRD: powder X‐ray diffraction

## 1 INTRODUCTION

Crystallization from a solution has been used in a great variety of scientific and industrial fields as efficient methods for structural determination and concentrating and purifying chemicals [1, 2]. In crystallization, various solution conditions such as super‐saturation, temperature, solvent composition, and pH affect nucleation and growth, as well as crystal quality [3, 4]. Furthermore, it is known that flows and interfaces work effectively for crystallization. For examples, crystallization of proteins and small organic molecules that are pharmaceutical compounds is promoted by a flow and gas‐liquid interface by laser or ultrasonic irradiations [5, 6, 7, 8, 9, 10]. In this study, we report the crystallization of acetaminophen using the liquid–liquid interface phenomenon by a microchannel.

Acetaminophen (paracetamol) is a widely used antipyretic and analgesic drug, and also used as a model compound for crystallization research, especially polymorphic control [11]. Various polymorphs of acetaminophen have been reported, form I (stable phase) [12], form II (metastable phase) [13], form III (metastable phase) [14], and three hydrates (monohydrate [15], dehydrate [16], and trihydrate [17]). Form I is used in commercial formulations. Form II has the favorable properties of being more soluble and more readily compressible into tablets than form I. Recently, we reported the crystallization of the unstable phases by femtosecond laser and ultrasound irradiation [8, 18–20].

Microfluidics possess the potential to change experimental approaches in sciences and engineering [21, 22, 23, 24, 25, 26, 27, 28]. In mixing solutions, microscale flow mixing is very different to normal bulk‐scale mixing. It is characterized by a larger surface‐to‐volume ratio, and laminar rather than turbulent flow, generating well‐defined microenvironments [29, 30, 31]. Co‐flowing fluids in a microchannel form a liquid–liquid interface, where their molecular components (solvent and solute molecules) diffuse into each other rapidly, being the concentration gradients. In contrast, in a batch, the solution mixing process is uncontrolled and only the final composition of solute–solvent mixture is considered. The antisolvent crystallization of glycine using a supersaturation gradient in a microchannel has been reported [32]. To achieve complete solution mixing within the channel, a long channel (350 mm) was used, ensuring a sufficiently long residence time for the flowing stream (>10 s). The slow and long flowing process unfortunately resulted in the crystals formed attaching to the channel wall, leading to channel blockage [32]. Here, we examine the effect of short‐time liquid–liquid interface morphology due to flow rate changes on crystallization of acetaminophen using a short channel (120 mm). In the short channel we used, solution is not fully mixed in order to maintain the interface. This method produces crystals in the vial after the flow path. The microchannel needs a small amount of sample, generates a well‐stable microenvironment, and can be mechanized as a high‐throughput method. Therefore, it will be useful in the production of active pharmaceutical ingredients, proteins, fine chemicals, and nanocrystals.

**PRACTICAL APPLICATION**

This study showed that the microflow system is effective for crystallization and is able to contribute to polymorphism control. Because the microchannel needs a small amount of sample and can be mechanized, it is useful in the production of pharmaceuticals in the future. Furthermore, since microchannels of various shapes can be designed, various crystallization methods using the microflow system can be expected.

## 2 MATERIALS AND METHODS

### 2.1 Microflow system

**FIGURE 1:**
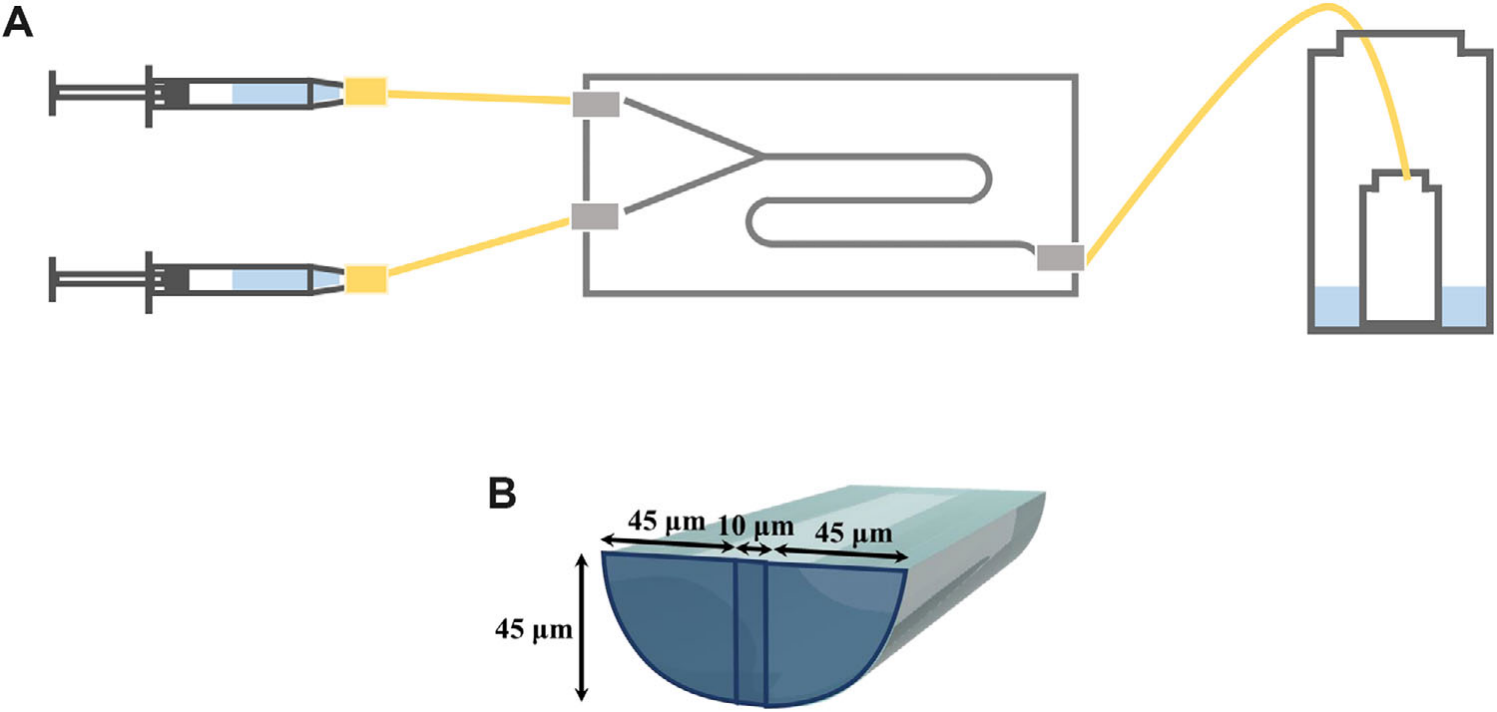
Experimental setup of microflow system. (A) Whole image. (B) Cross‐section of the microchannel

Figure 1 shows a schematic drawing of the microflow system in this research. A glass‐made microchip was purchased from IMT (Institute of Microchemical Technology Co., Ltd.) as a custom tip. Two streams are designed to meet at a Y‐type cross‐point with a channel depth and width of 45 and 100 µm, respectively. The total channel length is 120 mm. Glass syringes are placed on syringe pumps and connected to the aluminum jigs by Teflon capillaries (length: 100 mm; inner diameter: 260 µm). The outlet of the microchip is connected to the Teflon capillary and the solution is eluted into a glass vial in a closed environment equilibrated with the final composition of solute–solvent mixture. After confirming a steady flow, the solution is collected in a vial and subjected to crystallization and observation. At the flow rate of 10 µL/min (46 mm/s), the passage time in the channel is less than 3 s.

### 2.2 Crystallization

Acetaminophen was purchased from Acros with a purity of 98%. This material was confirmed as being form I by powder X‐ray diffraction (PXRD) previously [19, 20]. Acetaminophen solutions with concentrations of 128.7 and 20.2 mg/mL were prepared by dissolving acetaminophen form I in the solvent (water/acetonitrile [25 w/w %]) and the anti‐solvent (water), respectively. The solutions were heated at 60°C for 3 h in a drying oven. After filtration (0.22 µm), the solutions were placed in an incubator at 55°C for 1 h and then cooled to 20°C at a constant rate of 3°C/h. After the constant cooling, they were maintained at that temperature for 1 day. The supersaturation of form x, σ_x_, was calculated using the formula σ_x_ = (*C* – *C*_x_)/*C*_x_, where *C* is the acetaminophen concentration and *C*_x_ is the solubility of form x. *C*_I_ was estimated by using the values obtained from a previous paper [18, 33]. The supersaturations of solutions prepared here for form I at 20°C were σ_I_ = 0.3 (solvent) and 0.6 (anti‐solvent). Under each solution condition, no spontaneous crystallization occurred. After mixing these solutions, the supersaturation was σ_I_ = 1.0. As a control, bulk mixing of these solutions in a vial was carried out at 20°C. Obtained crystals were observed under an inverted microscope. The polymorphs were identified by PXRD measurements, as described previously [19]. The crystallization probability was calculated as (*n*_cry_/*n*_total_) × 100, where *n*_cry_ and *n*_total_ are the number of samples crystallized and the number of total samples examined, respectively.

## 3 RESULTS

### 3.1 Solution mixing in the microchannel system

**FIGURE 2:**
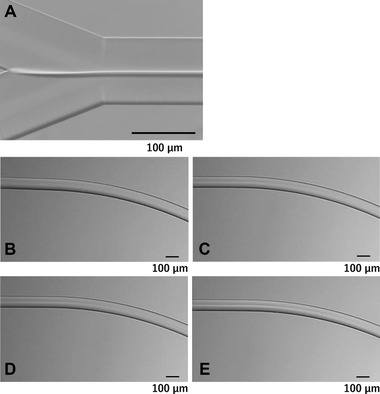
Photo images of the flow channel for the mixing process of the solvent (upper; water/acetonitrile [25 w/w %]) and the anti‐solvent (lower; water). (A) At the cross‐point at a flow rate of 50 µL/min. (B‐E) Near the first curve at about 30 mm after the cross‐point at the flow rate of (B) 10, (C) 25, (D) 50, and (E) 100 µL/min

**FIGURE 3:**
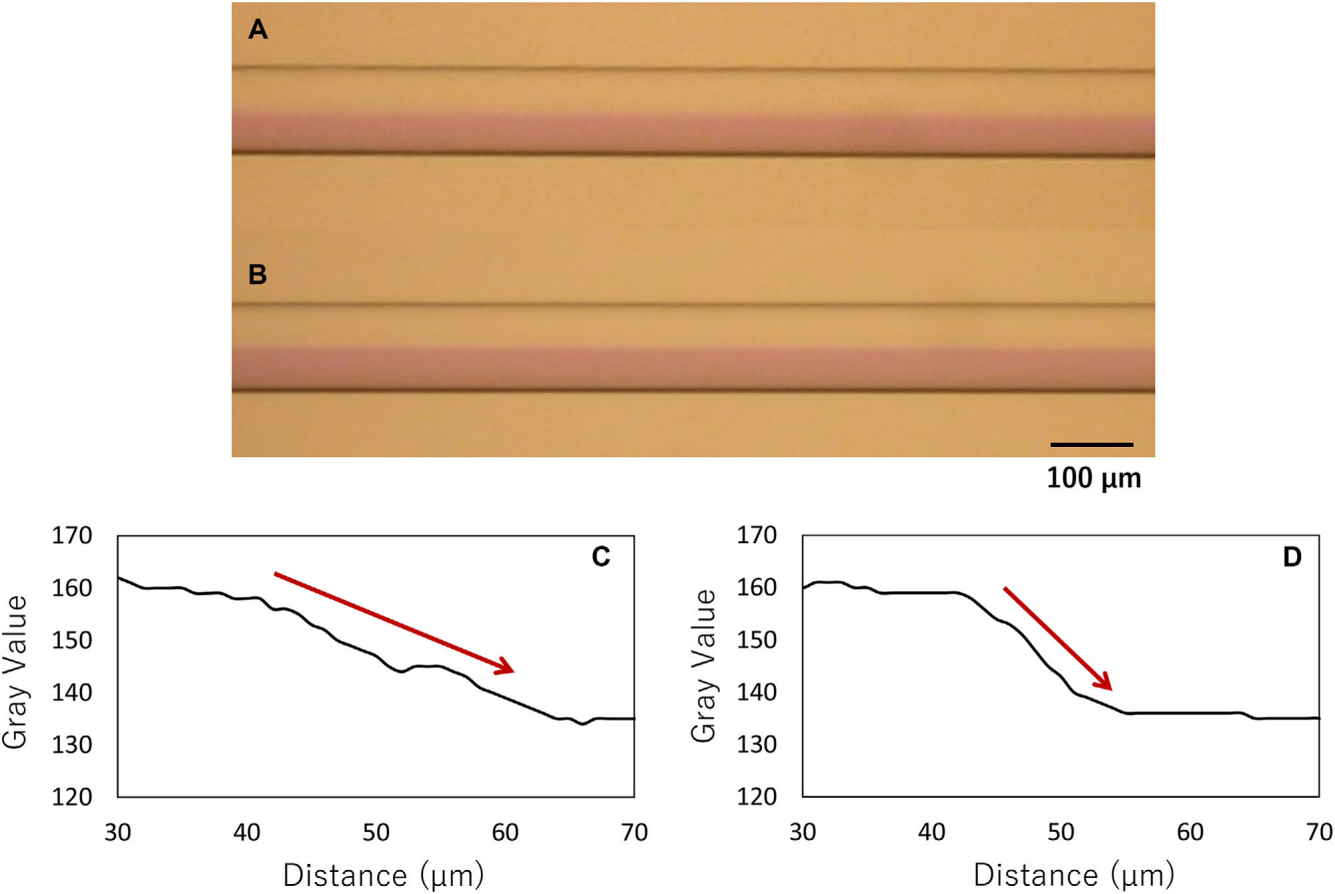
(A‐B) Photo images of the flow channel at about 10 mm after the cross‐point for the mixing process of the solvent (upper; water/acetonitrile [25 w/w %]) and the anti‐solvent (lower; water) colored with CBB at the flow rate of (A) 10 and (B) 100 µL/min. (C‐D) The color changes in the cross‐sectional direction of the flow channel determined using ImageJ at the flow rate of (C) 10 and (D) 100 µL/min

Before the crystallization of acetaminophen by the anti‐solvent method using the microchannel, the mixing process of the solvent (water/acetonitrile [25 w/w %]) and the anti‐solvent (water) in this microchannel system was observed. Figure 2A shows the cross‐point at the flow rate of 50 µL/min. After mixing the solutions, an interface was formed between the two solvents. Figures 2B–E represent the images near the first curve at about 30 mm after mixing with changing flow rates. In the flow from left to right, the interface at the left end is clearly visible in the order of E, D, C, and B, that is, in the order of flow rate. Furthermore, the higher the flow rate, the more the interface was maintained. We then observed using an anti‐solvent colored with CBB. At the lower flow rate of 10 µL/min, the interface was apparently blurrier than at the higher flow rate of 100 µL/min (Figure 3A,B). The color change in the cross‐sectional direction of the flow channel was determined using ImageJ [34]. As shown in Figure 3C,D, the higher the flow rate (100 µL/min), the higher the gradient of color changes at the interface. These results indicate that as the flow rate increases, the interface is maintained at a distance, and when the flow rate decreases, the solution mixing proceeds in the channel. Therefore, by changing the flow rate, the concentration gradients of solute and anti‐solvent in the microchannel can be changed.

### 3.2 Crystallization of acetaminophen

**FIGURE 4:**
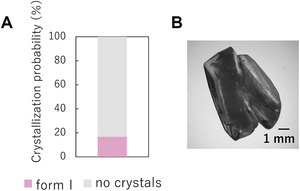
(A) Crystallization probability and (B) photo image of acetaminophen crystals by bulk mixing

**FIGURE 5:**
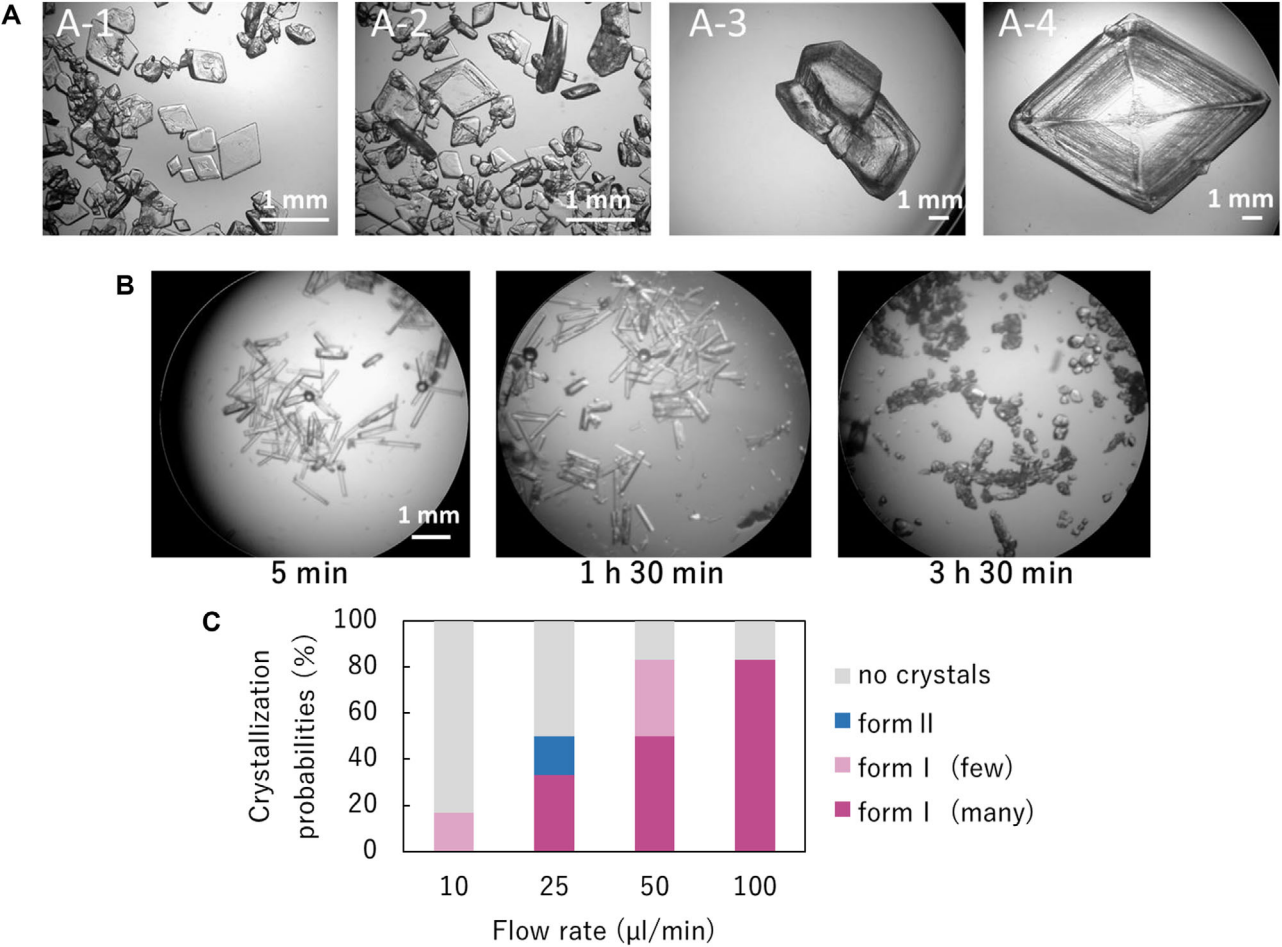
(A) Photo images of acetaminophen crystals (A‐1; 50 µL/min, A‐2; 100 µL/min, A‐3; 10 µL/min, A‐4; 50 µL/min). (B) Photo images of the phase transition process from acetaminophen form II to form I. (C) Crystallization probabilities by the microchannel mixing

In the bulk mixing, the crystallization probability was low (17%), and only a few form I crystals were obtained after 1 day (Figure 4). The result shows that the solution condition used here in the anti‐solvent method was severe even for form I crystallization. Next, crystallization of acetaminophen was attempted using the microchannel at 20°C. Acetaminophen in the solvent and anti‐solvent was mixed in the microchannel by changing different flow rates. No crystallization was observed within the microchannel, and crystallization was performed in a vial after the channel. Figure 5A represents the obtained crystals. In some vials, a large number of crystals appeared immediately, and in others only a small number of crystals were obtained after a couple of hours. The most crystals except for those in one vial at the flow rate of 25 µL/min were plate‐like and identified as form I by PXRD measurement. The crystals firstly appeared in the vial at the flow rate of 25 µL/min were prism‐like and transformed into another polymorph around 200 min after crystallization (Figure 5B). The crystals after the transition could be identified as form I, but the PXRD measurement of the crystals before the transition was not possible. In our previous work, prism‐like crystals were identified as form II and the transition to form I was observed [18]. In addition, a metastable to stable transition is known from crystal polymorphism theory [11]. Therefore, the pre‐transition crystals were presumed to be form II. This indicates that metastable crystals can be produced during crystallization in microchannels.

The crystallization probabilities were estimated by classifying them into many/few crystals, and polymorphisms (Figure 5C). Crystallization was promoted more in the faster flow than in the cases of lower flow and bulk mixing. These results indicate that the microflow mixing is effective for promotion of crystallization and selective crystallization of polymorphs.

## 4 DISCUSSION

For solution mixing in a microflow system, if the flow rate is higher, the liquid–liquid interface is maintained at a longer distance. However, the solution stays in the channel for a short time because the flow is fast. In the present results, fast flow promoted crystallization. This suggests that maintaining the interface and forming a supersaturation gradient is more advantageous for crystallization than mixing the solution well in the channel due to slow flow. The solvent and anti‐solvent with low supersaturation become high supersaturation at their interface [35], and the concentration gradient covers the nucleation conditions by varying degree of supersaturation. This may be related to the phenomenon whereby a surface always tries to minimize its energy. This can be done by adsorbing molecules with a lower energy onto its surface [36]. The molecules collected at the interface may also be regularly oriented depending on the properties of the solvents [37].

In the microchannel experiments, form II crystals were uniquely observed at the flow rate of 25 µL/min. The appearance of form II suggests that the liquid–liquid interface formed a high concentration region locally, resulting in the nucleation of form II. The transition of form II to form I indicates that not only form II but also form I nucleated, and the nucleation of form I occurred after form II. This is because the nucleation rate of form I is slower than that of form II [11]. In the fast flow where crystallization was promoted, form II was not observed. It is considered that the form II generated in an early stage had already undergone a phase transition with a large number of form I appearing later. These results suggest that the microchannel can be used for polymorphic control by setting the conditions such as solution concentration and flow rate. How to set them for polymorphic control is a future subject.

## CONFLICT OF INTEREST

The authors have declared no conflict of interest.
